# Multi-gate Weighted Fusion Network for neuronal morphology classification

**DOI:** 10.3389/fnins.2024.1322623

**Published:** 2024-11-08

**Authors:** Chunli Sun, Feng Zhao

**Affiliations:** MoE Key Laboratory of Brain-inspired Intelligent Perception and Cognition, University of Science and Technology of China, Hefei, China

**Keywords:** weighted fusion, hierarchical descriptors, morphological representation, multiple views, neuronal morphology analysis

## Abstract

Analyzing the types of neurons based on morphological characteristics is pivotal for understanding brain function and human development. Existing analysis approaches based on 2D view images fully use complementary information across images. However, these methods ignore the redundant information caused by similar images and the effects of different views on the analysis results during the fusion process. Considering these factors, this paper proposes a Multi-gate Weighted Fusion Network (MWFNet) to characterize neuronal morphology in a hierarchical manner. MWFNet mainly consists of a Gated View Enhancement Module (GVEM) and a Gated View Measurement Module (GVMM). GVEM enhances view-level descriptors and eliminates redundant information by mining the relationships among different views. GVMM calculates the weights of view images based on the salient activated regions to assess their influence on the analysis results. Furthermore, the enhanced view-level features are fused differentially according to the view weight to generate a more discriminative instance-level descriptor. In this way, the proposed MWFNet not only eliminates unnecessary features but also maps the representation differences of views into decision-making. This can improve the accuracy and robustness of MWFNet for the identification of neuron type. Experimental results show that our method achieves accuracies of 91.73 and 98.18% on classifying 10 types and five types of neurons, respectively, outperforming other state-of-the-art methods.

## 1 Introduction

Identifying and analyzing the types of neurons based on morphological features is essential for understanding brain function and development (Parekh and Ascoli, 2013; Colombo et al., 2022), as well as their links to brain diseases (Mages et al., 2018; Llorens-Mart́ın et al., 2016; Caznok Silveira et al., 2024). The complexity and variability of neuronal morphology make neuron type classification exceptionally challenging (Rapti, 2022; Weis et al., 2021). Numerous studies are committed to comprehensive representations of neuronal morphology and facilitate accurate analysis of neuron types. Traditionally, these methods are conducted by computing predefined morphometrics from the 3D neuron data (Mihaljević et al., 2018; Batabyal et al., 2020; Li, 2021; Batabyal et al., 2018; Lin et al., 2018) or automatically extracting deep features from the 2D view images (Li et al., 2018; Zhang et al., 2021).

**Figure 1:**
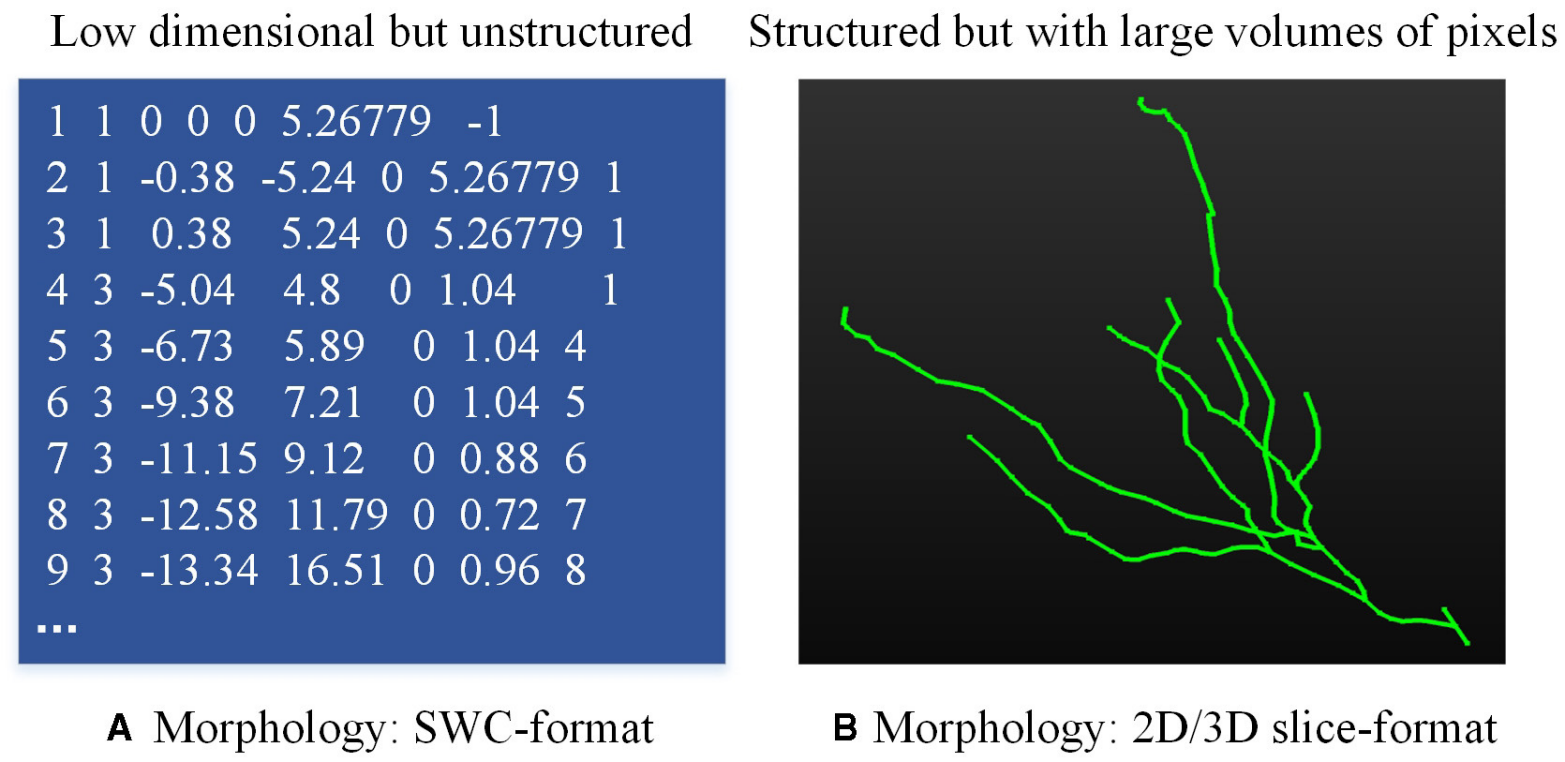
Neuronal data in different formats. **(A)** is the neuron data stored in the SWC file and **(B)** is the 2D/3D slice image.

Neuronal morphologies are typically recorded in the SWC format file (Stockley et al., 1993), which is a low dimension yet unstructured file (as shown in Figure 1A). The neuron is compressed as the structure of an un-strict binary tree (Gillette and Ascoli, 2015; Gillette et al., 2015). Given these characteristics, many methods (Mihaljević et al., 2018; Batabyal et al., 2020; Li, 2021; Wan et al., 2015; Batabyal et al., 2018; Zhang et al., 2019; Vasques et al., 2016) are developed to compute predefined morphometrics (Polavaram et al., 2014; Uylings and Van Pelt, 2002; Scorcioni et al., 2008; Bird and Cuntz, 2019), such as the number of branches, the number of compartments, the neuronal width, height, and depth, as well as the Euclidean distance of a compartment to soma. These predefined morphometrics are then fed into traditional classifiers or neural networks to analyze and identify neuron types (Lin et al., 2018; Yamashiro et al., 2021; Lin and Zheng, 2019). Furthermore, some research concentrates on the representation of the topological structure of neuronal branches (Gillette and Ascoli, 2015; Gillette et al., 2015; Hernández-Pérez et al., 2019; Kanari et al., 2018), which simplifies the representation of neurons into the 1D data. While these methods provide promising performance at some tasks, predefined features fall short when characterizing neurons with highly complex dendrites or axons (Fogo et al., 2021; Zhao et al., 2022). To fully represent neuronal morphology, some researchers turn to utilize 2D/3D neural networks to automatically and directly extract deep features from 3D neuron data instead of predefined morphometrics (Lin and Zheng, 2018; Zhu et al., 2023, 2022). However, utilizing 3D neural networks to analyze neuron types based on morphological characteristics presents several challenges. Firstly, the sparsity of 3D neuron data degrades the performance of these methods. Secondly, it requires more computing resources and time to train 3D neural networks. Thirdly, since there are significant differences in the number of nodes of neurons, it is hard to design a unified framework for processing 3D neuron data. Therefore, how to represent neuronal morphology with appropriate data format is the basis of type analysis.

To address the limitations of analyzing neuronal morphology using 3D networks, recent advances shift focus to analyze neural morphology utilizing 2D view images and neural networks (Li et al., 2018; Zhang et al., 2021; Schubert et al., 2019; Li et al., 2021). In their works, neurons are depicted through 2D view images, projected from one or more points of view (as shown in Figure 1B). Besides, some methods capture more distinct descriptors for neuronal morphology by directly combining the features extracted from different 2D images and predefined morphometrics, and then the combined features are fed into the classifier to predict the neuron types (Li et al., 2018). Notably, Li et al. (2018) proved that the deep features obtained from multiple images and predefined morphometrics are complementary in describing neuronal morphology. Furthermore, Zhang et al. (2021) first extract features from 3D neuron data and 2D images using tree–based recurrent neural network (TRNN) and convolutional neural network (CNN), respectively. These features are then directly fused to generate a comprehensive descriptor. Their experimental results confirm that employing the merged feature descriptor can produce better classification results than using either feature alone. These methods take advantage of the complementarities among view images or different features to improve classification performance, but they do not consider the differences in characterizing neuronal morphologies and information overlap across view images. Since 2D images may sometimes present similar neuronal morphologies, there is a risk of retaining redundant information in the morphological descriptors of neurons. Moreover, the importance of different view images varies, since they characterize different neuronal morphologies. Therefore, improving classification performance by concatenating the features of views differentially during the fusion process remains a desirable goal.

**Figure 2:**
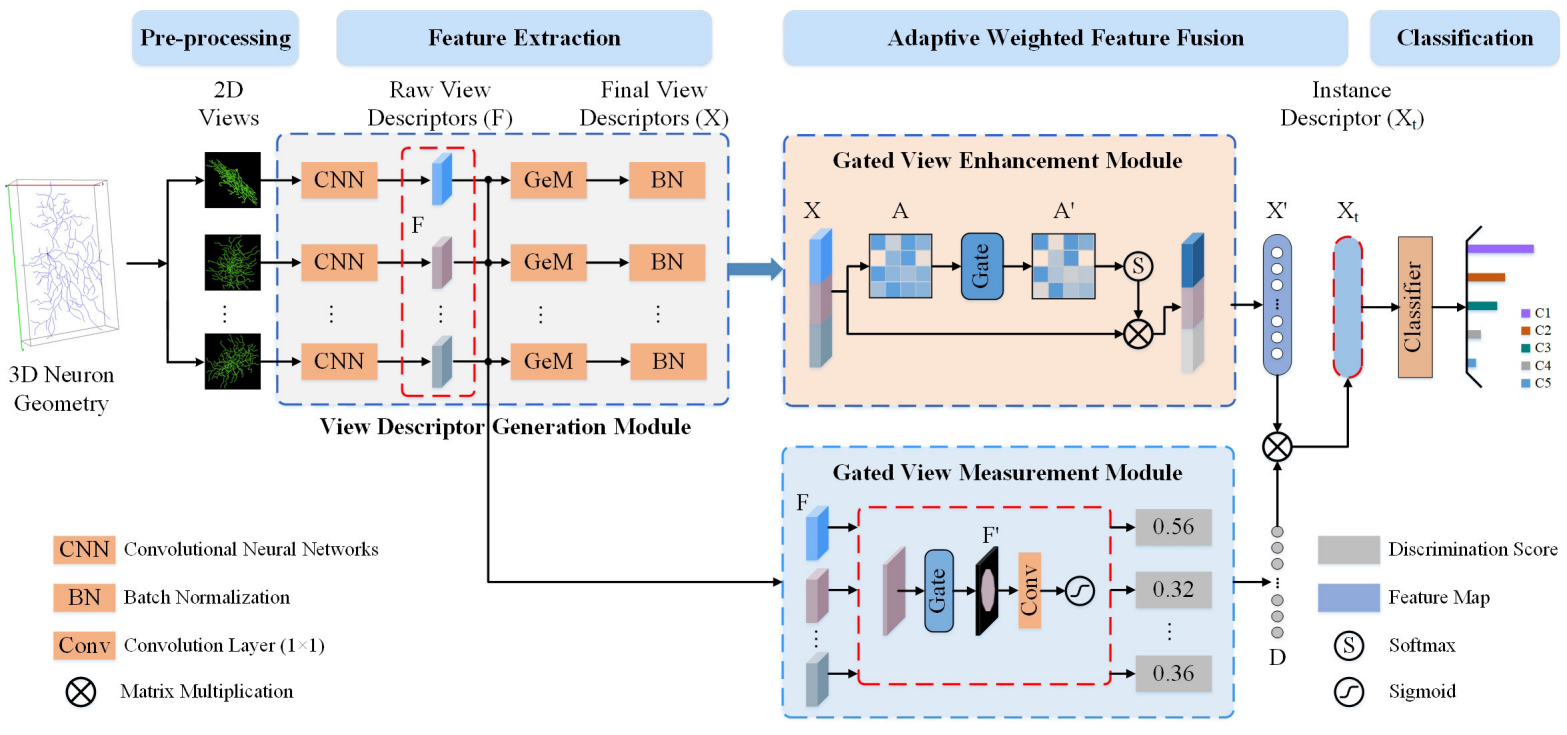
The proposed Multi-gate Weighted Fusion Network (MWFNet) with multiple views. First, multiple 2D view images are obtained from the 3D neuron geometry. Then, these images are fed to CNNs with Generalized-Mean (GeM) pooling to extract raw view-level descriptors (*F* and *X*). Next, the discriminability score (*D*) of *F* is computed via the Gated View Measurement Module (GVMM) and the enhanced view-level descriptors (*X*′) is obtained from *X* using the Gated View Enhancement Module (GVEM). Then, the instance-level descriptor (*X*_*t*_) is derived by adaptively assigning *D* to the *X*′. Finally, the instance-level descriptors *X*_*t*_ is fed to a classifier to predict neuron type.

To leverage the complementary information across 2D view images while accounting for information redundancy and morphological differences, we present a Multi-gate Weighted Fusion Network (MWFNet) with multiple views (shown in Figure 2). MWFNet builds more effective deep feature descriptors for neuronal morphology in a hierarchical manner, treating different view features in a differentiated way. Specifically, a Gated View Measurement Module (GVMM) is developed to measure the influence of each view on classification decisions. It computes the discriminability scores of different views only based on the salient activation regions, accounting for the sparsity of neuron data. Additionally, a Gated View Enhancement Module (GVEM) is implemented to explore the relationship between views and enhance the view-level descriptors, utilizing different views more efficiently while removing redundant information. Finally, the instance-level descriptors of neurons are obtained by adaptively assigning associated discriminability score to individual view descriptor. In this way, a neuron can be effectively characterized by the novel fused deep features, which capture the distinctness and distinguishability of view images while containing less redundant information.

The primary contributions of this work are given as,

- Our proposed MWFNet hierarchically establishes more efficient feature descriptors for neuronal morphologies. The MWFNet tactfully reduces the redundant information across 2D images and simultaneously measures the representation difference among the view images.
- We provide a Gated View Measurement Module (GVMM), which quantifies the impact of different views on analysis results by generating discriminability scores from the salient activation regions.
- We propose a Gated View Enhancement Module (GVEM) that mines the relationship between view images to enhance the distinct view descriptors and remove redundant information.
- We report superior classification accuracies, including 91.73% on the 10-type dataset and 98.18% on the 5-type dataset, outperforming other methods.

## 2 Methodology

As shown in Figure 2, the proposed MWFNet characterizes neuronal morphology in a hierarchical manner. View-level features are first extracted from the 2D view images using the CNNs, and then the instance-level descriptors of neurons are obtained via adaptively concatenating these view-level features. MWFNet mainly consists of four modules, namely data preprocessing, view descriptor generation, GVMM, and GVEM. During the data preprocessing, 3D neuron data is transformed into multiple 2D view images. Subsequently, view-level descriptors of these view images are generated via CNNs with Generalized-Mean (GeM) pooling. Next, the GVMM quantifies the discriminability of each view based on the represented neuronal morphology, while the GVEM enhances the view feature and reduces redundant information among views by mining the relationship between them. Finally, the enhanced features learned from different views are adaptively combined to obtain instance-level descriptors that comprehensively characterize the neurons.

### 2.1 Data preprocessing

Directly training unified and suitable 3D neural networks with 3D neuron data is challenging due to the sparsity of neurons in the 3D space and the variety of neuronal structures (Li et al., 2018). While networks based on multi-view projection (Su et al., 2015; Feng et al., 2018; Hamdi et al., 2021) and voxel- and point cloud-occupancy (Wu et al., 2015; Qi et al., 2017; Zhang et al., 2023) are used for 3D shape analysis, projection-based models can match the performance of 3D networks. In our initial attempts, we attempt to categorize neurons directly using 3D networks based on voxelized or interpolated 3D neuron data. It yields the accuracy of 63.78 and 64.45% on the 5-type dataset, respectively. These classification performance is unsatisfactory and can be further improved. Therefore, motivated by the success of projection-based models in 3D objection shape recognition (Su et al., 2015; Feng et al., 2018; Hamdi et al., 2021), we attemp to project the 3D neuron data into multiple binary images from various angles to address the challenges posed by varying neuronal structures and the sparse nature of 3D neuron data.

**Figure 3:**
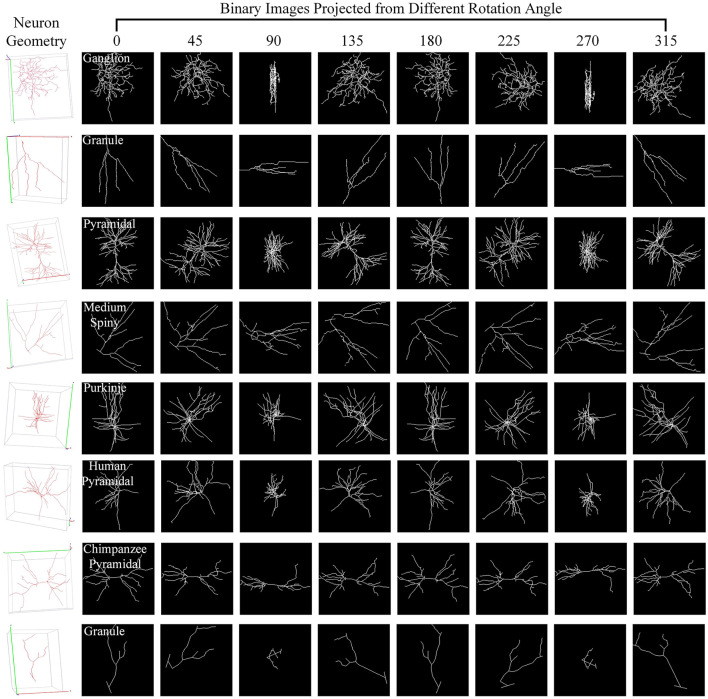
Neuron samples and their corresponding 2D images projected from multiple points of view. Here, each 3D neuron sample (shown in the first column) is projected into eight 2D view images (shown in columns 2–9), with a projection interval of 45 degrees. The types of neurons are indicated by the white text labels.

For one 3D neuron sample (*V* = {*v*_1_…, *v*_*n*_}) consisting of *n* nodes, we first utilize the principal component analysis (PCA) to align it along a normalized axis, as done in Li et al. (2018), Li et al. (2021), and Sun et al. (2023). This ensures that similar 2D view images are obtained from similar 3D neurons, regardless of their original orientation. We then project the 3D neuron points into 2D images from various angles of view to minimize the information loss and capture more neuronal morphology details. To obtain more 2D view images and additional view information, we rotate the neuron data along the *y*-axis by an angle θ and then rotate it around the *z*-axis by the same angle θ. Here, we simply set the rotation angle along the *y*-axis and *z*-axis the same. Since the morphological information along the *z*-axis is relatively limited, we use the *x*–*y* plane as the projection plane to reserve as many morphological characteristics as possible. The projection angle θ is incremented at ϕ intervals within the range [0°, 360°). In this way, each neuron sample can be projected into *N* 2D view images. For details, the projection processing is shown in Algorithm 1. It is worth noting that the raw 3D neuron data only contains information on multiple discrete points of a neuron. Each point is linked to its parent point to reflect the connectivity among discrete points in the neuronal morphology. Both the points and linked lines are depicted to present and preserve the morphology of neuronal arbors in projected binary images (as shown in Figure 3). Subsequently, these view images are fed into 2D neural networks to effectively build informative feature representations.

**Algorithm 1:**
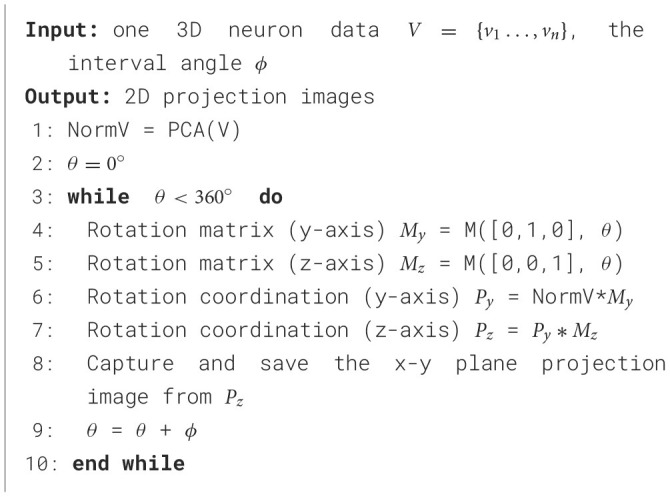
Generation 2D view images from 3D neurons.

### 2.2 View descriptor generation module

The proposed MWFNet framework extracts deep features at both view and instance levels to comprehensively characterize neurons. Given a set of view images projected from the original 3D neuron data, each view passes through a CNN to construct the raw feature map (*F*∈*R*^*C*×*H*×*W*^) at the view level, as shown in Figure 2. Then, following the approach of Ye et al. (2021), we adopt the learned GeM pooling (Radenovic et al., 2019) to capture the view-specific discriminative features, given by,


(1) $$\begin{array}{l} \begin{array}{c} x={\left[f_{1},f_{2},\cdots \;,f_{c},\cdots \;,f_{C}\right]}^{T},\\ f_{c}={\left(\frac{1}{\left|\chi_{c}\right|}\sum_{x_{i}\in \chi_{c}}x_{i}^{p_{c}}\right)}^{\frac{1}{p_{c}}}, \end{array} \end{array}$$

where *C* is the number of the feature map of the last convolutional layer of the CNN, χ_*c*_ denotes the *c*-th feature map in the *F*(*c*∈{1, 2, ⋯ , *C*}), and *f*_*c*_ represents the result of the χ_*c*_ after pooling. The pooling hyper-parameter *p*_*c*_ can be kept constant or continuously optimized during the training (Radenovic et al., 2019; Deng et al., 2019). Notably, GeM pooling becomes average pooling when *p*_*c*_ is 1 or max pooling when *p*_*c*_ approaches ∞. In practice, the performance of GeM pooling is better than that of max pooling or average pooling in morphology-based neuron classification. A batch normalization (BN) layer is used to normalize the features to a unified data space, facilitating the fusion of features from different views.

### 2.3 Gated View Measurement Module (GVMM)

Different views affect the classification results differentially due to the varying morphological information they represent (as shown in Figure 3). Based on this, we build the GVMM to measure the differences between view images, efficiently learning the neuronal morphologies embedded within the projected view images. For each view, we then use the GVMM calculates a discrimination score as view weight to adaptively aggregate features into an distinguishing neuron representation. Taking into account the sparsity of neuron data and limited neuronal morphology in the view images, we construct a masked view descriptor. This seeks to identify the critical neuronal morphologies through the salient feature regions, not the background or noise region. To this end, we mask view descriptors below the threshold value (*T*_1_) by multiplying a binary mask (*M*). The masked view descriptors (*F*′) can be formulated as,


(2) $$\begin{array}{l} F_{ij}^{\prime }=F_{ij}*M_{ij},\\ M_{ij}=\{\begin{array}{c} 1,\;if\;F_{ij}\geq T_{1}\\ 0,\;if\;F_{ij}<T_{1} \end{array} \end{array}$$

where *i* and *j* are the row and column of the raw view descriptors *F*, respectively. The masked view descriptors activate the most pertinent neural regions while suppressing the background and low-reliability feature regions.

We then calculate the distinguishability of views based on this masked view descriptors (*F*′). First, a convolution layer with a kernel size of 1 × 1 is employed to reduce the dimensionality of the masked view descriptors to the half of the number of channels. Next, a flatten operation, a bath normlization layer and a fully connected (FC) layer are applied. The dimensionality of the output of FC layer is 1. Subsequently, the discriminability of each view is quantified by a function *V*(·), defined as,


(3) $$d=V\left(F^{\prime }\right)=\delta \left(\log\left(\left|F^{\prime }\right|\right)\right),$$

where δ is the *sigmoid* function and |·| is used to calculate the absolute value of *F*′. When the input of the *sigmoid* function is < –5 or >5, its output will be close to 0 or 1 accordingly. Thus, the |·| and *log* functions are added before the *sigmoid* function to avoid this situation.

### 2.4 Gated View Enhancement Module (GVEM)

**Figure 4:**
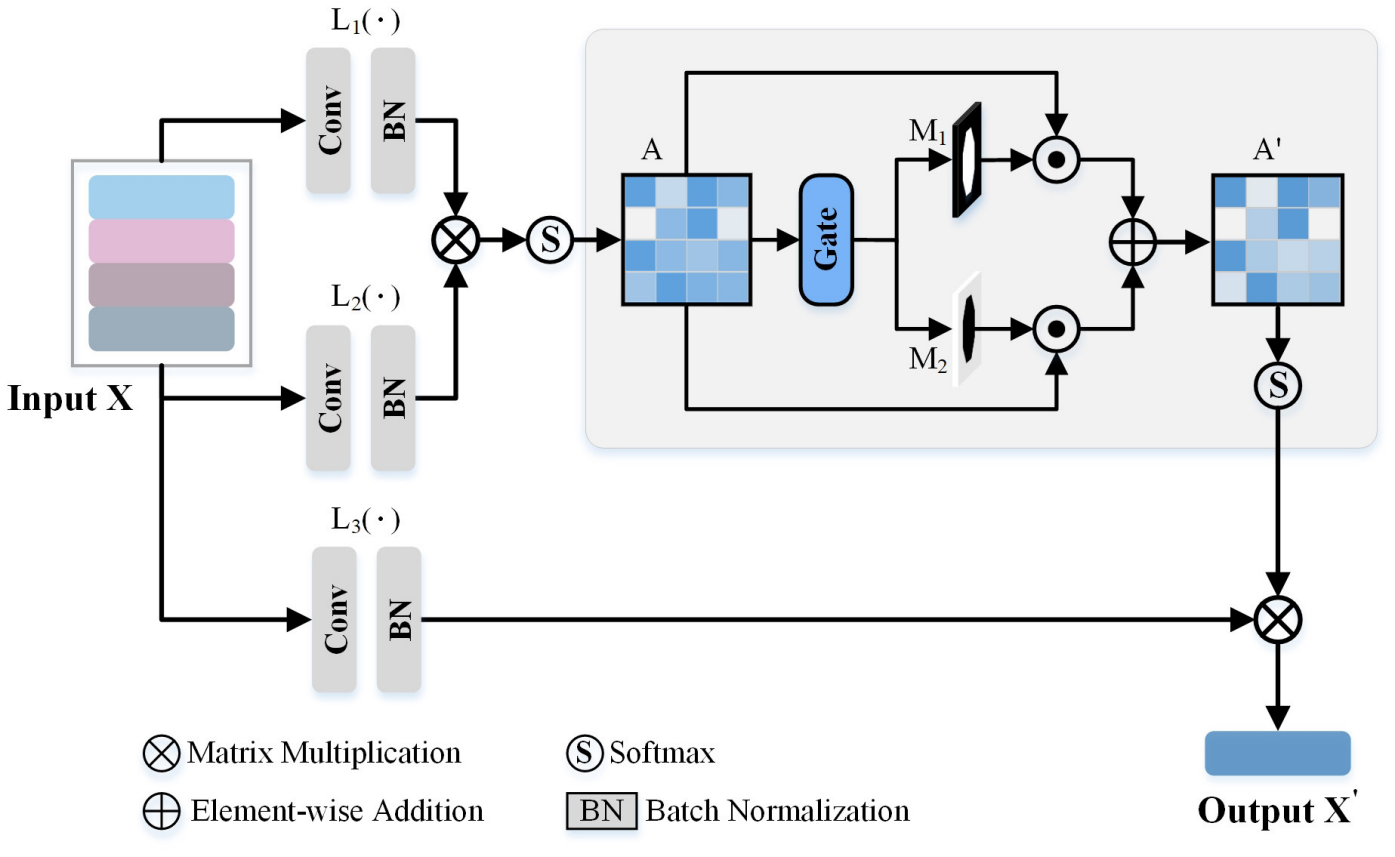
The Gated View Enhancement Module (GVEM). It first calculates the paired similarity matrix (*A*) between the view features obtained from the feature transform layers [*L*_1_(·) and *L*_2_(·)]. Based on *A*, two masks (*M*_1_ and *M*_2_) are generated to respectively select the paired views with higher and lower similarity. Finally, the modified paired similarity matrix (*A*′) is generated and used to weaken the paired view features with high similarity while enhancing the paired view features with low similarity.

As observed from Figure 3, some views exhibit similarities, with overlapping morphological information across view images. Consequently, we introduce a GVEM (as shown in Figure 4) to fully leverage the morphological information represented by different views while reducing redundancies introduced by similar views. The GVEM explores the relationships between different views and utilizes these relationships to guide the enhancement of view-level descriptors.

The extracted view-level descriptor (*X*∈*R*^*N*×*C*×1 × 1^) after GeM pooling is utilized to obtain enhanced view features based on the pairwise similarity matrix. Three feature transform layers [*L*_1_(·), *L*_2_(·), and *L*_3_(·)], consisting of a 1 × 1 convolution layer and a normalization layer, are employed to transform different view descriptors into a unified space. Subsequently, the similarity matrix (*A*) is attained based on the pairwise relations *a*_*i, j*_∈[0, 1] between view features, where *a*_*i, j*_ is computed as:


(4) $$\begin{array}{l} a_{i,j}=\frac{exp\left(L_{1}{\left(x_{i}\right)}^{T}\cdot L_{2}\left(x_{j}\right)\right)}{\underset{j}{\sum }exp\left(L_{1}{\left(x_{i}\right)}^{T}\cdot L_{2}\left(x_{j}\right)\right)}, \end{array}$$

where exp[*L*_1_(*x*_*i*_), *L*_2_(*x*_*j*_)] measures the similarity between the features of *i*-th and *j*-th view images. *L*_1_(*x*_*i*_) and *L*_2_(*x*_*j*_) are the outputs of the first two feature transform layers. Note that the *exp*, namely exponential function, is employed to enlarge the differences between different view descriptors.

Since the difference between the two view descriptors may be relatively small, leading to potential redundant information in the final neuron instance descriptor, we utilize the gate-based module to generate two selective masks. These masks are employed to reasonably adjust the feature values of different views, further enhancing the view descriptors. In more detail, we use a threshold *T*_2_ to conduct two binary masks, which are formulated as,


(5) $$\begin{array}{l} \begin{array}{c} M_{1}=I\left(A\geq T_{2}\right),\\ M_{2}=I\left(A<T_{2}\right), \end{array} \end{array}$$

where *I* is the logical matrix, and the shape is the same as *A*. *M*_1_ and *M*_2_ will be 1 when the *A* is greater than *T*_2_ and lower than *T*_2_, respectively. Based on these two masks, we modify the paired similarity matrix by,


(6) $$A^{\prime }=\frac{1}{\exp(A)}⊙M_{1}+\exp\left(A⊙M_{2}\right).$$

Then, the *softmax* function is utilized to normalize the enhanced similarity matrix. Through the above process, we reduce the ratio of the view features with high similarity in the enhanced view descriptors while amplifying the view features with low similarity.

The enhanced view descriptors are obtained as the product of the learned view relation matrix *A*′ and the output of *L*_3_(*X*), denoted by,


(7) $$X^{\prime }=\;A^{\prime }*L_{3}\left(X\right),$$

where *X* is the view descriptors after the GeM pooling, the *L*_3_ is the third feature transformer layer, and the *A*′ is the masked relation matrix of views. By this approach, information redundancy is reduced, and the impact of view features comprising more distinct morphology information is increased.

### 2.5 View fusion module

This study uses a weighted-view fusion strategy with residual batch normalization to exploit the view-level descriptors properly and train the MWFNet robustly. The instance-level descriptor of one neuron is then obtained by combining the enhanced view descriptors generated by the GVEM based on the corresponding view weights produced by the GVMM, which is formulated by,


(8) $$X_{t}=DX^{\prime },$$

where *D* = {*d*_*n*_|*n* = 1, 2, ⋯ , *N*} is the learnable view weight calculated by the GVMM, and *X*′ is the enhanced view descriptors obtained from the GVEM. Finally, the instance-level descriptors of neurons are fed into the classifier to predict their types. Here, the classifier includes two FC layers and a softmax layer. Each FC layer reduces the feature dimension to 200. The predicted class for neurons results from the softmax layer with the highest probability.

## 3 Experimental results

In this section, we present a comprehensive evaluation of the proposed MWFNet through a series of experiments. First, we introduce the validation dataset and the experiment setups. We then report the performance of our method and compare it with other existing methods. Additionally, the robustness and generalization capabilities of the MWFNet are verified in this section. Finally, we conduct ablation experiments to analyze the influence of the individual components of the proposed MWFNet on the final classification performance.

### 3.1 Datasets

NeuroMorpho.org (Ascoli et al., 2007) is one of the largest web-accessible repositories for digitally reconstructed neurons. It contains 183,740 neurons (version 8.2.36) from multiple brain regions, species, and types, contributed by over 900 laboratories worldwide. It is important to note that the quality of digitally reconstructed neurons varies. While some neurons contain complete dendrites and axons, others only have partial dendrites or lack axons entirely. Moreover, neurons of different types can exhibit similar morphologies, while neurons of the same type can differ in morphology (Li et al., 2021). This makes the identification of neurons more challenging. To ensure an objective and fair comparison between our method and existing methods, we randomly selected all neuron data from NeuroMorpho.org (Ascoli et al., 2007) without considering the physical integrity of neurons.

#### 3.1.1 Dataset 1

As presented in Table 1, this dataset consists of 10 classes of neurons belonging to multiple brain regions and species. It contains pyramidal cells (from the chimpanzee, mouse, rat, and human), pyramidal cells from rat neocortex and hippocampus, as well as rat GABAergic and nitrergic interneurons. Besides, granule and medium spiny neurons belonging to various species are also considered. There are 500 neurons for each type.

**Table 1:** The considered neuron classes of Dataset 1.

**ID**	**Neuron class**	**Number**
C1	Chimpanzee principal pyramidal	500
C2	Various principal granule	500
C3	Human principal pyramidal	500
C4	Various principal medium spiny	500
C5	Mouse principal ganglion	500
C6	Mouse principal pyramidal	500
C7	Rat interneuron gabaergic	500
C8	Rat interneuron nitrergic	500
C9	Rat principal pyramidal hippocampus	500
C10	Rat principal pyramidal neocortex	500

#### 3.1.2 Dataset 2

To verify the generalization of our method, the dataset only considering neuronal type also serves as a validation dataset. It consists of 1,802 digitally reconstructed neurons belonging to five classes, namely ganglion, granule, motor, Purkinje, and pyramidal cells. The number of samples per class is 500, 500, 95, 208, and 499, respectively. For clarity, these five neuron types are denoted as GA, GR, MO, PU, and PC, respectively.

### 3.2 Experiment settings

#### 3.2.1 Implementation details

In the data prepossessing, the ϕ is set to 45°. Consequently, the number of view images *N* is 8, and each view image is resized to 224 × 224 pixels. In the view descriptor generation module, the ResNet-50 is employed to extract raw view features. The learnable GeM pooling parameter *p*_*c*_ is initialized to 3. Besides, *T*_1_ and *T*_2_ in GVMM and GVEM are set as 0.9 and 0.8, respectively. The MWFNet framework is implemented using PyTorch and two NVIDIA GTX 2080Ti GPUs are employed to train the model with a batch size of 16. The Adam optimizer with a learning rate of 0.001 is adopted to optimize the cross-entropy loss, while other parameters are set to their default values.

Besides, we employ the 10-fold cross-validation method to assess the performance of our MWFNet. Therefore, the dataset is randomly divided into 10 equal-sized folds. In each validation iteration, one fold serves as the test set while the remaining nine serve as the training set. This process is repeated 10 times, ensuring that each subset is used as a test set. Consequently, our MWFNet is trained on 10 distinct training sets and evaluated on 10 separate test sets. Finally, we calculate the average of these 10 validation results to estimate the overall performance of our MWFNet.

#### 3.2.2 Evaluation metrics

Overall accuracy is naturally utilized to evaluate the classification performance of our method. Besides, the *F*_1_ score serves as an evaluation metric to verify the effectiveness of the proposed approach. Classification confusion matrices are also provided to clearly demonstrate the effectiveness of our method.

### 3.3 Classification performance of MWFNet

**Figure 5:**
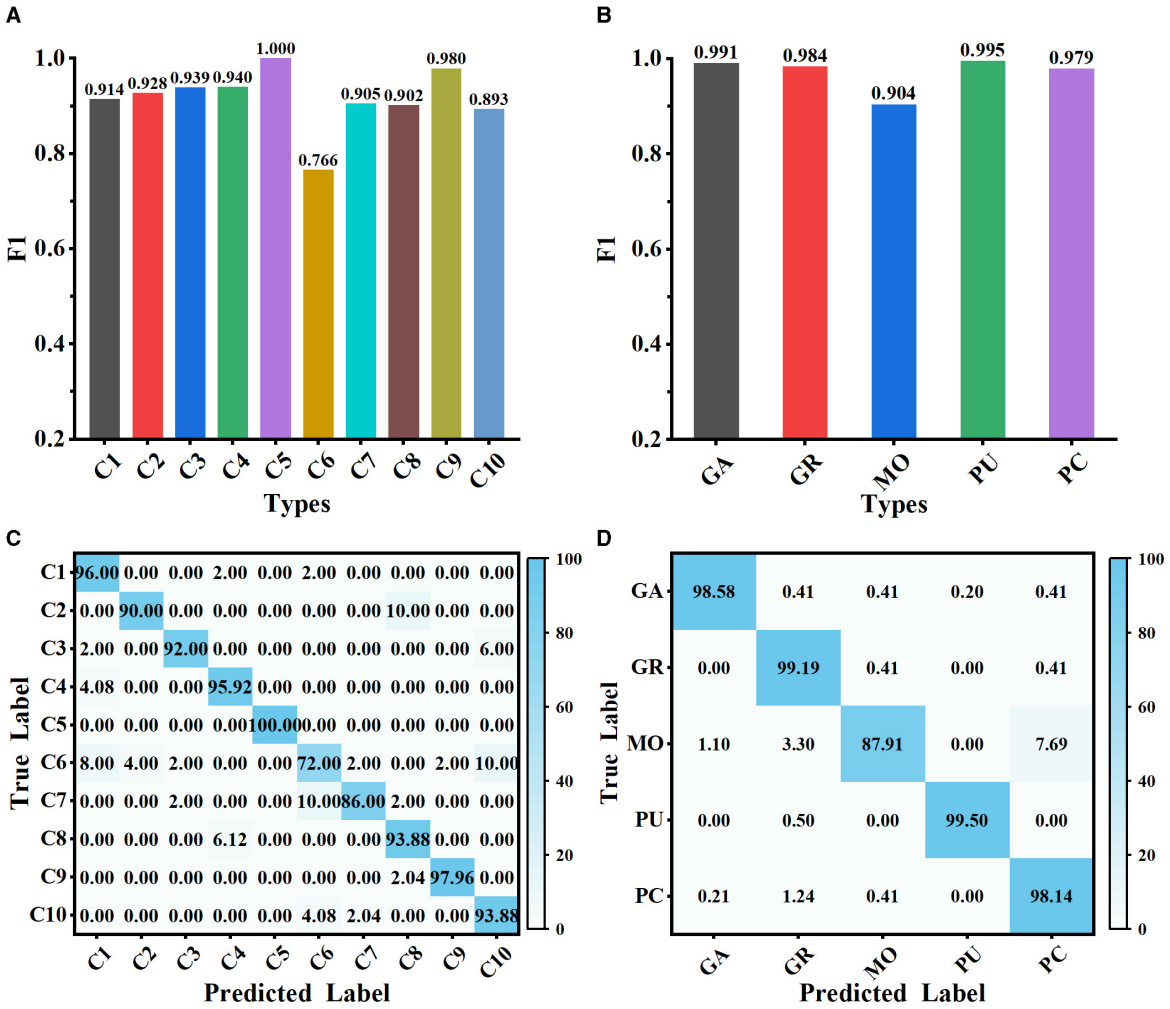
Classification performance of MWFNet on both datasets. **(A, B)** are the *F*_1_ scores of each type evaluated on Dataset 1 and Dataset 2, respectively. **(C, D)** are the confusion matrices of classification on Dataset 1 and Dataset 2, respectively.

The proposed method is evaluated on Dataset 1 and Dataset 2, and the classification performance is shown in Figure 5. Our method achieves satisfactory results on the 10-class classification task, with an average accuracy of 91.73% and an *F*_1_ score value of 0.917. It is noteworthy that the *F*_1_ scores of all classes on Dataset 1, except C6, exceed 0.890, implying that the extracted deep features can effectively represent the differences among neuron classes (as shown in Figure 5A). We observe that our method has some limitations in distinguishing rat pyramidal cells (i.e., C6 type), only with an *F*_1_ score of 0.766. 10% of mouse pyramidal cells are misclassified as rat pyramidal cells (i.e., C10 type; as shown in Figure 5C). There are some morphological similarities between these pyramidal cells, such as apical dendrites, basal dendrites, and soma. While our method is good at capturing the overall structure of neuronal morphology, it currently has some limitations in identifying subtle morphological nuances, such as dendrite size and branching patterns. Our future work will focus on improving the ability of our method to represent subtle differences so that it can accurately describe the morphological properties of neurons both globally and locally. When evaluated on Dataset 2 with 5 neuron types, our method exhibits better classification performance with an average *F*_1_ score of 0.971 (as presented in Figure 5B). The confusion matrices report a high true prediction rate for neuron types (as shown in Figures 5C, D), indicating that our method can precisely distinguish neurons with the instance-level descriptors.

**Figure 6:**
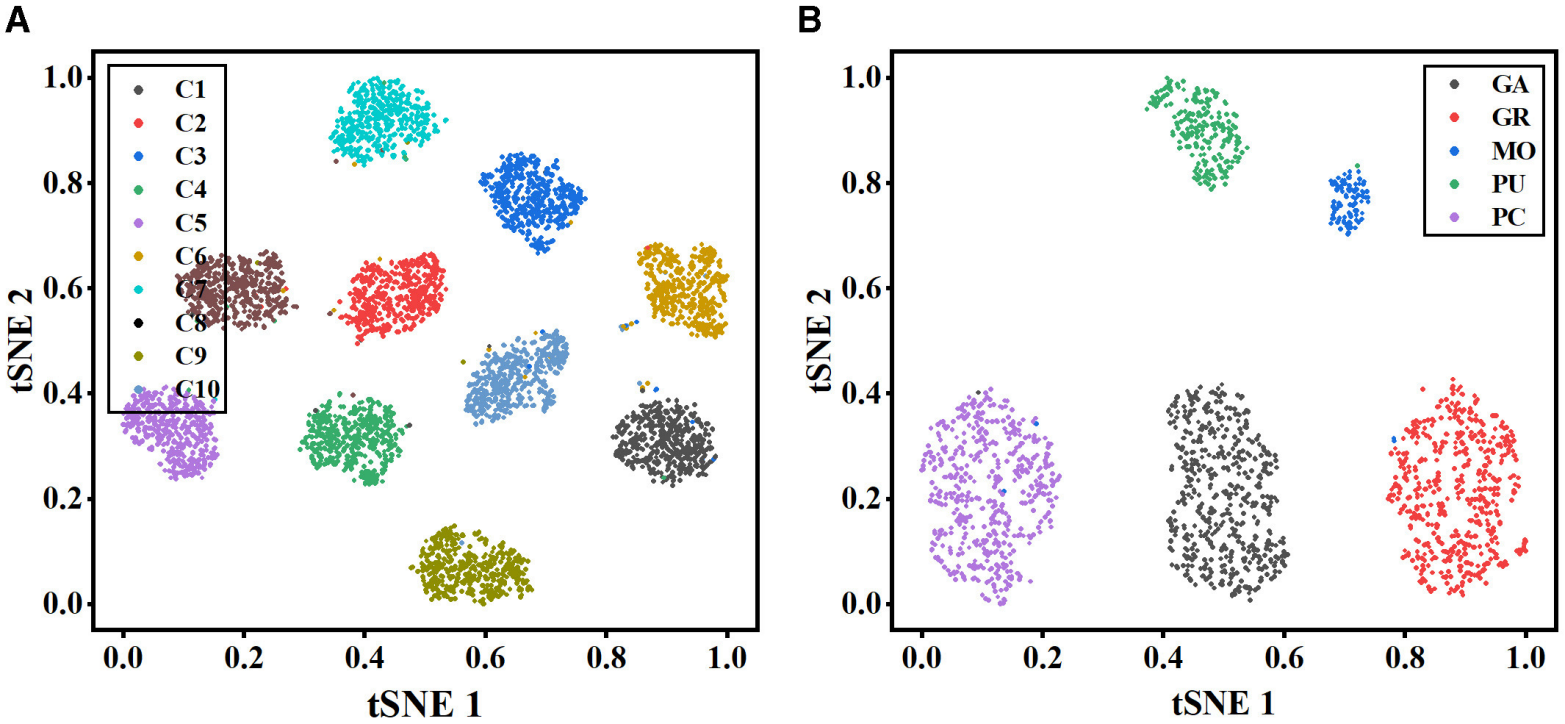
Visualization of the instance-level feature vectors using the t-SNE. **(A, B)** are the feature distributions of Dataset 1 and Dataset 2, respectively.

To further illustrate the effectiveness of our method, t-stochastic neighbor embedding (t-SNE) (Van der Maaten and Hinton, 2008) is employed for the analysis of the distribution of high-dimensional features. Figure 6 depicts the distribution of the instance-level features extracted from the MWFNet on both datasets. Despite the great inter- and intra-class variation in neuronal morphology, our method clearly separates each category into a distinct cluster. By enhancing the raw view feature and reducing redundant information among views, the instance-level features learned from the MWFNet contribute to the accurate identification of different neurons. Both visual and qualitative experimental results demonstrate the superiority of our method in neuronal morphology analysis.

### 3.4 Comparison with other methods

We compare our method with various methods evaluated on two datasets to demonstrate its efficacy, including Morphometrics (Vasques et al., 2016), 3D CNN (Lin and Zheng, 2018), DRNN (Lin et al., 2018), LCCDNN (Lin and Zheng, 2019), TRNN (Zhang et al., 2021), SCAEs (Li et al., 2018), and TreeMoCo (Chen et al., 2022). Especially, the Morphometrics is evaluated by using 43 predefined morphometrics computed by L-Measure (Scorcioni et al., 2008) as feature descriptors. Then, various supervised algorithms and training settings are utilized as the previous work (Vasques et al., 2016), and the best performance is reported. Additionally, we evaluate the performance of our method, using only three orthogonal views (namely *x*–*y*, *y*–*z*, and *x*–*z* planes) projected from the 3D neuron data as input. This configuration is referred to as MWFNet-3 Views. For other methods, to make a fair comparison, we train these models using the same dataset and train-test split as our method. Moreover, we utilize the default parameter of these compared models to optimize them. The best results are reported.

As presented in Table 2, the proposed method yields the best classification performances with an accuracy of 91.73% and an *F*_1_ score of 0.917 on Dataset 1. Our method has a striking accuracy improvement by 10.45% over Morphometrics (Vasques et al., 2016). This indicates that the novel feature representations produced by the MWFNet are more effective at characterizing neurons with complex morphology structures. Compared with the 3D CNN (Lin and Zheng, 2018) based on the voxelized 3D data, our method makes full use of the complementary information across views while enhancing the view-level feature of each view to compensate for the information loss caused by projection. Therefore, our method can comprehensively depict neuronal arbors and precisely identify the type of neurons. By considering the impact of different views on the classification results, our method gains a 6.73% accuracy improvement compared with TRNN (Zhang et al., 2021), which directly connects different view features to generate the instance-level descriptor. Although TreeMoCo (Chen et al., 2022) constructs the neuron as a tree graph and designs many features, it involves neuron node sampling during the data preprocessing, so its performance can be further improved. Besides, MWFNet performs better when takes multiple views as input instead of three orthogonal projection views.

**Table 2:** Comparison of different methods.

**Method**	**Input**	**Dataset 1**		**Dataset 2**	
		*F*_1_	**Acc. (%)**	*F*_1_	**Acc. (%)**
Morphometrics (Vasques et al., 2016)	1D	0.803	81.28	0.859	88.33
DRNN (Lin et al., 2018)	1D	0.502	54.00	0.841	84.53
LCCDNN (Lin and Zheng, 2019)	1D	0.534	55.20	0.855	85.64
SCAEs (Li et al., 2018)	2D	0.623	62.76	0.664	73.48
3D CNN (Lin and Zheng, 2018)	3D	0.429	45.82	0.605	63.78
TreeMoco (Chen et al., 2022)	3D	0.753	75.70	0.922	92.19
TRNN (Zhang et al., 2021)	2D+3D	0.836	85.00	0.950	94.00
MWFNet-3 Views (Ours)	2D	0.881	88.20	0.962	96.69
MWFNet (Ours)	2D	**0.917**	**91.73**	**0.971**	**98.18**

The best results are highlighted in bold.

Table 2 also reports the classification performance of different methods evaluated on Dataset 2. Our method achieves an accuracy of 98.18% and an *F*_1_ score of 0.971 on Dataset 2, outperforming other methods on all evaluation metrics. Compared with the 3D CNN (Lin and Zheng, 2018), our method improves the classification accuracy by 34.4%. The information loss of part neuronal arbors caused by the voxelization and the sparsity of neuron data negatively affects the performance of 3D CNN (Lin and Zheng, 2018). Despite possessing an average *F*_1_ score of 0.950, the TRNN (Zhang et al., 2021) performs 2.1% worse than our method. This is because it ignores the distinctions between view features and their influence on analysis outcomes. Conversely, our approach measures the discriminability of various view images and treats view features differentially. Consequently, our method can effectively improve the performance of neuron classification.

### 3.5 Validation of robustness

The proposed MWFNet is evaluated on the dataset, randomly downloaded from NeuroMorpho.org (Ascoli et al., 2007). It is a comprehensive database, contributed to by over 900 laboratories worldwide, presenting a wide array of experimental conditions and data quality. To thoroughly assess the capabilities of MWFNet, diverse selection criteria based on neuronal dendrites are utilized to generate the evaluation dataset. Specifically, 10 types of neurons within Dataset 1 are first categorized based on the physical integrity of their dendrites, labeled as complete, moderately complete, or incomplete. Subsequently, they are organized into multiple sub-datasets: neurons only with complete dendrites (**C**), neurons only with moderately complete dendrites (**M**), neurons only with incomplete dendrites (**I**), neurons with dendrites that are at least moderately complete (**C+M**), and a comprehensive group including all neuron types (**I+C+M**).

Table 3 shows the classification performance of the proposed MWFNet evaluated on these sub-datasets, evidencing its robust classification efficacy. Notably, our MWFNet attains an impressive accuracy of 94.36% in classifying neurons with complete dendrites (**C** dataset). For the **M** dataset, including neurons with moderately complete dendrites, MWFNet achieves an accuracy of 90.83%. Remarkably, it also yields an accuracy of 89.77% on the **I** dataset, despite the significant challenges involved in classifying neurons with exclusively incomplete dendrites. When evaluated on the **C+M** dataset, MWFNet achieves satisfactory performance with an accuracy of 92.00%. Crucially, it proves its comprehensive applicability by correctly identifying 91.73% of neurons across all categories. These findings confirm that MWFNet delivers high-quality analysis across neuron datasets with variable reconstruction quality, showcasing its reliability as an effective tool for large-scale and diverse neuron type analysis.

**Table 3:** Classification accuracy of our method evaluated on datasets with different physical integrity of neuronal dendrites.

**Dataset**	***F*_1_**	**Acc. (%)**
**I**	0.882	89.77
**M**	0.907	90.83
**C**	0.941	94.36
**C+M**	0.919	92.00
**I+C+M**	0.917	91.73

**I**, **M** and **C** denote the datasets only including neurons with incomplete, moderate, and complete dendrites, respectively. The **C+M** dataset includes neurons with moderate and complete dendrites. The **I+C+M** dataset includes neurons with incomplete, moderate, and complete dendrites.

### 3.6 Validation of generalization

**Figure 7:**
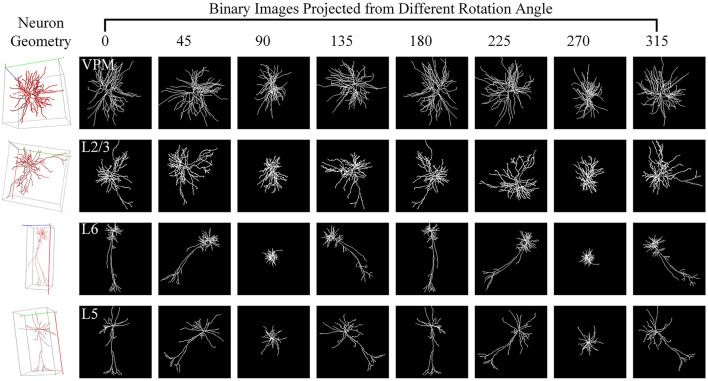
Neuron samples of the JML-4 dataset and their corresponding 2D images projected view images. Each 3D neuron sample (shown in the first column) is projected into eight view images (shown in columns 2–9), with a projection interval of 45 degrees. The types of neurons are labeled with white text labels.

Here, we utilize the digitally reconstructed neurons provided by the Janelia MouseLight (JML, http://mouselight.janelia.org/) to verify the generalization of our approach. We use the same dataset as Chen et al. (2022) to make a fair comparison and give the optimal results reported in Chen et al. (2022). We eliminate neurons that exhibit clear reconstruction errors. Furthermore, although some samples have both dendrites and axons, we simplify our analysis by retaining only the dendritic arbors as done in Chen et al. (2022). Consequently, the selected JML-4 dataset used in this section includes 369 neurons belonging to L2/3, L5, L6, and VPM. Each class consists of 64, 179, 114, and 12 neurons. We employ the same training-test split as the work (Chen et al., 2022) and utilize the data preprocessing method described in Section 2.1 to obtain the 2D view images (as shown in Figure 7) as the input of our method.

As shown in Table 4, our method yields the best results, with an accuracy of 71.88%, outperforming other methods. It should be noted that this dataset exhibits data imbalance, with the number of samples for each class ranging from 10 to 200, and it suffers from a shortage of overall training data, totaling only 369 samples. Furthermore, neurons of different types often share similar morphologies, as seen in the neurons from layers L5 and L6 (as illustrated in Figure 7). These factors make neuron classification particularly challenging, resulting in relatively low performance across all comparison methods applied to this dataset. Despite these challenges posed by this dataset, our method delivers robust results. MWFNet effectively captures the complex morphologies of neurons through multiple projection images and sophisticated feature representation, enhancing its efficacy even with limited data availability. Furthermore, the proposed MWFNet can be used to effectively identify neurons from various resources.

**Table 4:** Classification accuracy of different approaches evaluated on the JML-4 dataset.

**Method**	**Acc. (%)**
MorphoVAE (Laturnus and Berens, 2021)	40.00
TRNN (Zhang et al., 2021)	51.43
GraphCL (You et al., 2020)	54.29
TreeMoCo (Chen et al., 2022)	67.14
MWFNet (Ours)	**71.88**

The best results are highlighted in bold.

### 3.7 Ablation studies

To verify the importance of each module of the proposed MWFNet, we perform ablation experiments. Firstly, we verify the effectiveness of GVMM and GVEM, respectively. Next, we analyze the representation differences of different views and explore the impact of such differences on the final results. Finally, we examine the effect of using different thresholds in the GVMM and GVEM.

#### 3.7.1 Validation of different modules

Here, we first verify the influences of GVMM and GVEM. The baseline method adopts ResNet-50 and GeM pooling as the feature extractor, concatenating different view features directly to generate the instance-level descriptors of neurons (see the first row of performance in Table 5). In our method, the GVMM aims to calculate the importance of views based on salient feature regions, eliminating the influence of background regions. As shown in the Table 5, incorporating the GVMM improves the accuracy by 9.2% and 2.7% over the baseline on both datasets while the number of parameters of GVMM is almost negligible. The GVEM mines the relationships between different views and enhances the view-level descriptors, further reducing redundant information among views and improving the performance of our method. The introduction of GVEM improves the accuracy of our method by 9.48% and 3.04% on the two datasets, respectively. When introducing both GVMM and GVEM, the accuracies of our method evaluated on both datasets gain 11.53% and 3.15% over the baseline, respectively.

**Table 5:** Performance of MWFNet with different modules.

**GVMM**	**GVEM**	**Inc. of Param. (M)**	**Dataset 1**		**Dataset 2**	
			*F*_1_	**Acc. (%)**	*F*_1_	**Acc. (%)**
–	–	–	0.796	80.20	0.898	95.03
✓	–	0.002	0.893	89.40 (9.20↑)	0.975	97.73 (2.70↑)
–	✓	12.600	0.896	89.68 (9.48↑)	0.969	98.07 (3.04↑)
✓	✓	12.620	0.917	91.73 (11.53↑)	0.971	98.18 (3.15↑)

Inc. of Praram. represents the increment of parameters and the ↑ represents the improvement of accuracy (%).

#### 3.7.2 Validation of different views

**Figure 8:**
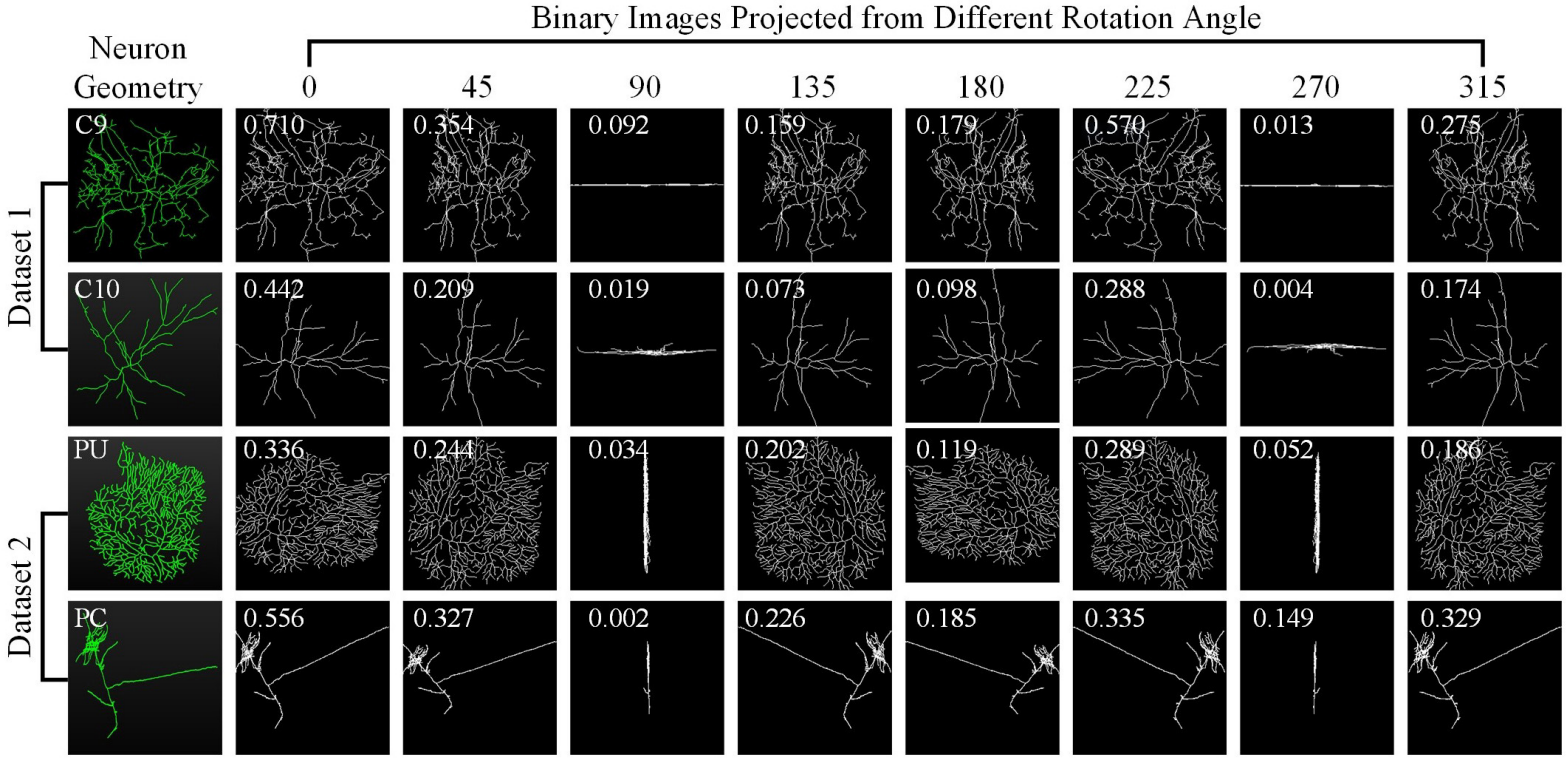
Neuron samples randomly selected from both datasets and the corresponding projected images. The discriminability score for each projected image is marked in white font.

Here, we investigate how each projection image affects the categorization results. Figure 8 shows the projected view images of neurons and the related weights produced by the GVMM. As presented in Figure 3, neurons exhibit comparable morphologies from the view images projected from the same projection angles. View images projected at different rotation angles show morphological distinctions. Additionally, the projected view images containing varying morphological details contribute differentially to the classification results. Here, the weight estimations of different views are shown in Figure 8 with white font. It is observed that the view image with more morphological details usually has a larger weight score, indicating that the view has more distinct morphological information and would strongly impact the classification decision, and vice versa. Therefore, it is sensible that our method treats the features from different views differentially by assigning adaptive weights in the fusion process. If the view-level features are treated equally, the instance-level descriptors of neurons are not sufficiently differentiated among different classes and may contain more redundant information, hindering effective neuronal morphology classification.

To further validate the efficacy of our method in treating different view images distinctly, we conduct experiments on Dataset 1 by manually assigning equal weights to view features. Table 6 presents the classification results of MWFNet with manual and adaptive weight assignment. It is evident that when each view is assigned the same weight manually (such as 0.2, 0.4, 0.6, 0.8, and 1.0), the classification performance is relatively poor, showcasing a notable disparity compared to the performance achieved by adaptively assigning weights computed by GVMM to different views. This highlights the effectiveness of our MWFNet in treating views differentially based on their morphology representation for neurons. Moreover, we also explore the impact of manually assigning varying weights to each view based on their representation differences in neuronal morphology. When setting corresponding weights to views, the results are comparable to the adaptive weight assignment. Interestingly, when opposite weights are manually assigned to views according to the morphology representation of each view for neurons, a slight decrease in classification performance can be observed. These results further demonstrate the validity of our method in utilizing the corresponding weights generated by GVMM for different view images.

**Table 6:** Classification accuracy of our method with different view weight assignment methods.

**Weights for 8 views**	**Setting method**	***F*_1_**	**Acc. (%)**
[0.2, 0.2, 0.2, 0.2, 0.2, 0.2, 0.2, 0.2]	Manual assignment	0.881	88.11
[0.4, 0.4, 0.4, 0.4, 0.4, 0.4, 0.4, 0.4]	Manual assignment	0.886	88.67
[0.6, 0.6, 0.6, 0.6, 0.6, 0.6, 0.6, 0.6]	Manual assignment	0.889	88.93
[0.8, 0.8, 0.8, 0.8, 0.8, 0.8, 0.8, 0.8]	Manual assignment	0.884	88.51
[1.0, 1.0, 1.0, 1.0, 1.0, 1.0, 1.0, 1.0]	Manual assignment	0.885	88.55
[0.1, 0.1, 0.55, 0.2, 0.5, 0.1, 0.5, 0.1]	Manual assignment	0.883	88.51
[0.5, 0.3, 0.01, 0.2, 0.1, 0.3, 0.1, 0.3]	Manual assignment	0.899	90.12
Adaptive weights generated by GVMM (Ours)	Adaptive assignment	**0.917**	**91.73**

There are eight view images and their corresponding view weights. All experiments are conducted on Dataset 1 and the best results are highlighted in bold.

#### 3.7.3 Threshold selection

Here, we explore the effects of different *T*_1_ and *T*_2_ for the GVMM and GVEM, respectively. All experiments are carried out on Dataset 1, consisting of more types of neurons.

**Figure 9:**
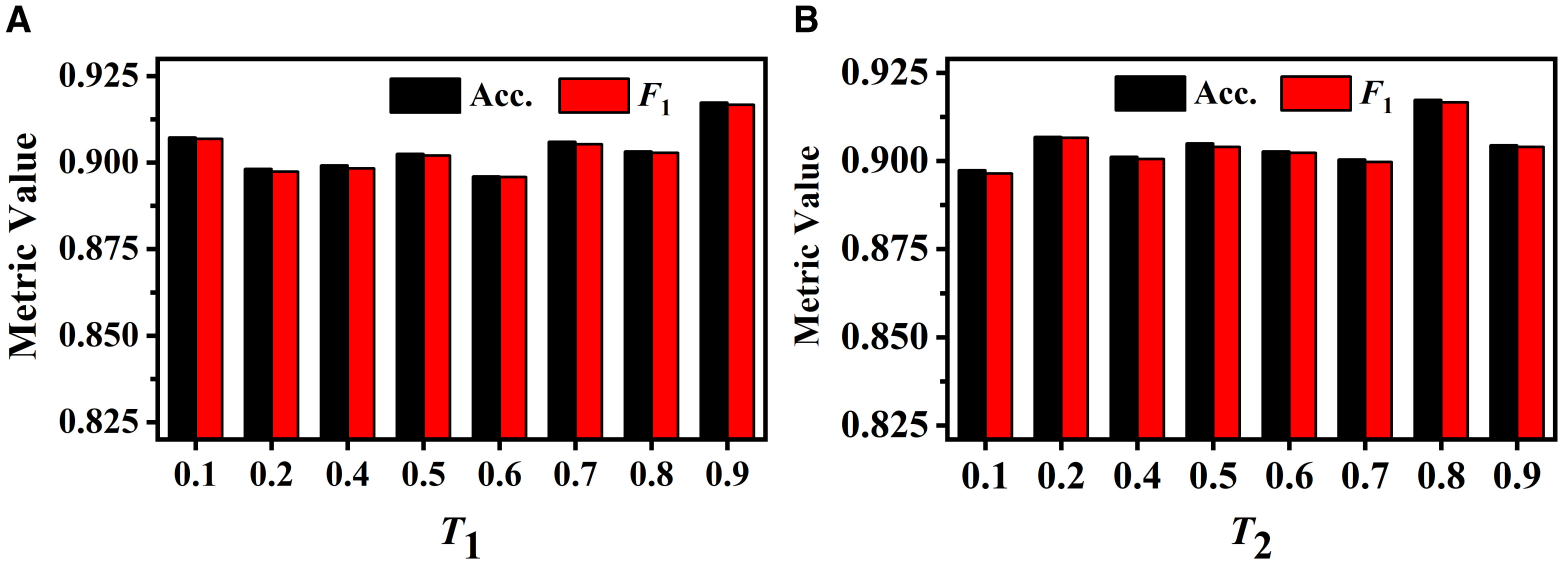
Performance of our method under different *T*_1_ on the GVMM **(A)** and under different *T*_2_ on the GVEM **(B)**.

We first investigate the influence of different *T*_1_ values on GVMM. We conduct experiments with different *T*_1_ to select the optimal value. As illustrated in Figure 9A, when the value of *T*_1_ is relatively small, the feature area used by GVMM to measure view confidence is wider. This makes the distinguishability measurement of each view less precise. To gauge the significance of the view for analysis results, GVMM selects high-confidence feature areas as the *T*_1_ value increases. This lessens the influence of background or noisy areas, allowing for improved categorization results. Our method yields the best performance across all three metric values when *T*_1_ is equal to 0.9. Therefore, we set the *T*_1_ to 0.9 for other trials.

The GVEM employs the threshold *T*_2_ to assess the similarity among the view images, reducing the weight of the highly similar view features while strengthening the less similar ones. Figure 9B displays the experimental results of our method based on various *T*_2_ values. We can observe that our method achieves the best classification performance when *T*_2_ is equal to 0.8. The highest performance (an *F*_1_ score of 0.917) presents a 2.02% gain compared to when *T*_2_ is 0.1. This demonstrates that GVEM can enhance view features and reduce redundancy, facilitating the learning of instance-level feature descriptors.

## 4 Discussions

Identifying and analyzing neuron types based on morphology are important to understanding the neuronal function and activity (Li et al., 2021; Parekh and Ascoli, 2013). However, it is challenging due to the significant differences in neuronal morphology among intra- and inter-classes (Li et al., 2021). Recently, automatic analysis methods (Zhu et al., 2022; Lin and Zheng, 2018, 2019) based on morphological characteristics mainly employ 3D CNNs or 2D CNNs to extract feature representations from 3D neuron data or 2D images, respectively. However, the sparsity of neuronal morphology makes it not easy to build a unified 3D network for various datasets (Li et al., 2018). While the method (Zhang et al., 2021) based on 2D images obtains a unified framework and saves computing resources, it does not account for the limitations and specificity of 2D views in characterizing neuronal morphology.

In this work, we propose the MWFNet, which hierarchically describes neuronal morphology based on multiple 2D view images. The MWFNet considers the differences between 2D views in representing neuronal morphology, as well as the similarity and repeatability among views. The obtained instance-level descriptors contain salient features learned from multiple-view images and reduce the redundant information induced from similar views. Therefore, our method can fully represent neuronal morphology and accurately reflect the characteristics of different categories.

As Figure 8 illustrates, different view images depict neuronal morphology differentially. Consequently, their influence on the analysis results varies. The GVMM employs threshold *T*_1_ to select high-confidence and salient feature regions to assess the impact of each view. Experimental results show that the GVMM effectively improves the performance of our method in identifying 10 types and 5 types of neurons. The ablation study on the selection of *T*_1_ shows that our method produces the best classification results (accuracy of 91.73% and 98.10% on Dataset 1 and Dataset 2, respectively) when the threshold *T*_1_ is equal to 0.9. However, the threshold *T*_1_ value is manually selected and set in this work. In future work, we will explore adjusting the *T*_1_ adaptively during the learning process to analyze the neuronal morphology conveniently.

We observe similarities between views (see Figure 8) when utilizing the multi-view method to describe neuronal morphology. However, if all view descriptors are used to form the instance-level descriptor, it leads to information redundancy. The GVEM is designed to improve the effectiveness of view-level features and retain dissimilar features. It enhances the feature representation of the views different from others while weakening the characteristics of these extremely similar views. According to experimental results, GVEM increases classification accuracy by 9.48% and 3.04% on Dataset 1 and Dataset 2, respectively. However, our method sets a high threshold *T*_2_ to retain as many view features as possible while removing redundant information to some extent. In our future work, we will consider removing redundant features to a greater extent while maintaining the classification performance of our method. Additionally, we will investigate setting the threshold *T*_2_ more flexibly.

## 5 Conclusions

This paper proposes a novel feature representation for neuronal morphology using the Multi-gate Weighted Fusion Network (MWFNet). The MWFNet first utilizes a Gated View Measurement Module (GVMM) to assess the impact of each view on the classification results according to the salient feature regions and a Gated View Enhancement Module (GVEM) to enhance view-level descriptors based on the paired similarity. After that, the discriminative instance-level descriptors for neurons are obtained by adaptively assigning the corresponding discrimination score generated by GVMM to the enhanced view features obtained from the GVEM. Experimental results show that our method achieves high accuracies of 91.73% and 98.18% on 10-type and 5-type neuron classification tasks, respectively, outperforming other methods. Moreover, the MWFNet has good generalization and robustness when evaluated on other datasets.

In the future, we will further optimize the proposed MWFNet and apply it to analyze large-scale datasets. While MWFNet yields significant performance, its GVMM and GVEM manually set thresholds. In future work, we will explore approaches to automatically adjust and set these thresholds during the feature extraction process. This will enable more automatic and scalable neuronal morphology analysis. Additionally, neuron data is increasing dramatically thanks to continuous advances in high-precision microscopic imaging and reconstruction techniques. Therefore, we plan to apply the proposed MWFNet to analyze larger-scale datasets. Our goal is to develop a robust and efficient tool for large-scale neuron analysis that will significantly advance the field of neuroscience.

## Data Availability

The original contributions presented in the study are included in the article/supplementary material, further inquiries can be directed to the corresponding author.
